# The “Perfect Trip” on social media: strategic self-presentation in travel sharing, posting anxiety, and retrospective trip satisfaction with social comparison orientation as a moderator

**DOI:** 10.3389/fpsyg.2026.1805325

**Published:** 2026-07-17

**Authors:** Weijun Zheng, Xianle Wang, Xia Chen, Wei Weng

**Affiliations:** 1 Faculty of Geography, Yunnan Normal University, Kunming, China; 2 International School of Technological Education, Sichuan College of Architectural Technology, Deyang, China; 3 School of Communication, Yunnan Normal University, Kunming, China; 4 Management School, Yunnan Normal University, Kunming, China

**Keywords:** moderated mediation, posting anxiety, self-presentation, social comparison orientation, trip satisfaction

## Abstract

**Background:**

Rooted in social psychological theories of self-presentation and social comparison, this study asks whether impression management in social media travel sharing is associated with affective costs that may relate to how people evaluate an otherwise positive life episode.

**Methods:**

Adults who had completed a leisure trip within the past three months and had posted travel-related content at least once completed an anonymous online survey (*N* = 465). Strategic self-presentation in travel sharing, social media posting anxiety, social comparison orientation, and trip satisfaction were measured with validated scales. Confirmatory factor analysis supported construct distinctiveness. We tested a moderated mediation model using latent moderated structural equations.

**Results:**

Strategic self-presentation was positively associated with posting anxiety, and posting anxiety was negatively associated with trip satisfaction. A small direct association between strategic self-presentation and lower trip satisfaction remained. Social comparison orientation moderated the strategic self-presentation-to-posting-anxiety association, such that the link was weak at low comparison orientation but markedly stronger at high comparison orientation. Conditional indirect effects showed that the negative indirect association between strategic self-presentation and trip satisfaction via posting anxiety increased as social comparison orientation rose.

**Conclusion:**

The findings highlight a psychological trade-off in online self-presentation. When travel sharing is guided by impression management, it may be associated with higher posting anxiety and slightly lower trip satisfaction, especially among individuals prone to social comparison.

## 1 Introduction

Social media posting has become a major contemporary venue for impression management. In everyday platform use, individuals do not simply record experiences; they often select, edit, and narrate experiences with an imagined audience in mind. This is especially visible in travel sharing, where leisure experiences are frequently transformed into public signals of lifestyle, taste, social connectedness, and personal identity. Classic self-presentation theory argues that people are motivated to control how they are perceived by others, particularly when desired impressions are important but uncertain (Schlenker and Leary, 1982; Leary and Kowalski, 1990). Social media intensifies this process because posts are persistent, editable, socially visible, and open to feedback from audiences that may be heterogeneous and only partly known to the content creator (Marwick and boyd, 2011). From this perspective, travel-related posting provides a theoretically rich context for examining how impression-management strategies may shape not only online behavior but also the psychological evaluation of an offline leisure experience.

The present study focuses on strategic self-presentation in travel sharing as a specific form of impression management. Strategic self-presentation refers to the tendency to curate travel-related content in order to create a desirable image, appear interesting or admired, and manage how one's trip is perceived by others. Importantly, such behavior is not assumed to be inherently harmful. Strategic presentation may help individuals communicate identity, preserve memories, maintain social ties, and obtain social recognition. However, impression-management theory also suggests that self-presentation may become psychologically costly when individuals perceive that the desired impression is difficult to achieve or vulnerable to negative evaluation (Schlenker and Leary, 1982; Leary and Kowalski, 1990). In social media contexts, this cost may appear as posting anxiety, namely apprehension about whether a post will be judged, misunderstood, ignored, or compared unfavorably with others' posts (Shabahang et al., 2022). Thus, the central question is not whether travel sharing is good or bad in general, but when audience-oriented self-presentation becomes associated with emotional strain and less favorable retrospective evaluation of the trip.

This question addresses an important gap in the literature. Prior research has shown that social media use has heterogeneous implications for wellbeing, depending on users' motives, activities, and interpersonal contexts (Valkenburg, 2022; Meier and Reinecke, 2021). Studies on social comparison further show that exposure to idealized portrayals of others can shape affective and self-evaluative outcomes (Appel et al., 2016; Verduyn et al., 2020; Meier and Johnson, 2022). In tourism research, social media sharing has often been discussed as a source of social value, memory construction, destination communication, and satisfaction (Munar and Jacobsen, 2014; Ghaderi et al., 2024; Wong et al., 2024). Yet, less is known about the psychological conditions under which travel sharing may backfire. In particular, existing work has not sufficiently explained how strategic self-presentation during or after a trip may be associated with posting-related anxiety, and how this anxiety may in turn relate to tourists' retrospective satisfaction with the travel experience.

To address this gap, we develop an impression-management framework that links strategic self-presentation, posting anxiety, social comparison orientation, and trip satisfaction. In this framework, strategic self-presentation represents an audience-oriented impression-management strategy; posting anxiety represents a proximal affective response to perceived evaluative risk; and social comparison orientation represents a dispositional tendency that may make audience evaluation and relative standing especially salient. Social comparison theory proposes that individuals evaluate themselves by comparing with others, especially when standards are ambiguous or socially relevant (Festinger, 1954). Individuals high in social comparison orientation are, therefore, more likely to interpret social media posting as a comparative and evaluative act (Gibbons and Buunk, 1999). Accordingly, the same degree of strategic self-presentation may be more strongly associated with posting anxiety among those who are chronically attentive to comparison information.

Trip satisfaction is examined as a retrospective evaluative outcome. We do not assume that tourists wait until they post on social media to form an opinion about their trip. Rather, we conceptualize post-trip satisfaction as an overall appraisal that may be associated with affective and cognitive processes surrounding the experience, including how the trip is narrated, evaluated, and socially presented. Research on documenting experiences suggests that capturing and sharing meaningful moments can influence attention, enjoyment, memory, and later evaluation, depending on whether documentation supports savoring and personal meaning or instead becomes dominated by external evaluation (Diehl et al., 2016; Diehl and Zauberman, 2022). In the present study, posting anxiety is expected to represent one psychological route through which strategic travel sharing may be linked to less favorable retrospective trip satisfaction.

The study makes three theoretical contributions. First, it extends impression-management theory by applying it to travel-related social media posting, a setting in which offline leisure experiences are transformed into public and evaluable self-presentations. This moves the theory beyond general online self-presentation by showing how impression-management concerns may be associated with the retrospective appraisal of a personally meaningful life episode. Second, it identifies posting anxiety as a specific affective mechanism through which audience-oriented self-presentation may become psychologically costly. This contributes to social media and wellbeing research by shifting attention from broad measures of social media use to a more precise psychological process tied to content creation and anticipated evaluation. Third, it specifies social comparison orientation as a boundary condition that helps explain for whom strategic self-presentation is more strongly associated with anxiety. In doing so, the study connects impression-management theory with social comparison theory and clarifies when self-presentation may shift from adaptive identity expression to emotionally costly evaluation management.

Based on this framework, we test a moderated mediation model using a cross-sectional survey of adult tourists who had recently completed a leisure trip and shared travel-related content on social media. Specifically, we examine whether strategic self-presentation in travel sharing is associated with lower retrospective trip satisfaction through higher posting anxiety, and whether the association between strategic self-presentation and posting anxiety is stronger among individuals with higher social comparison orientation.

## 2 Literature review and hypothesis development

**Figure 1:**
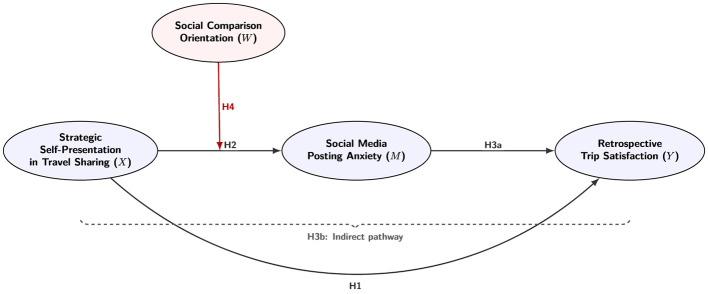
The proposed conceptual model. H1, Direct association; H2, Association between strategic self-presentation and posting anxiety; H3a, Association between posting anxiety and retrospective trip satisfaction; H3b, Indirect pathway through posting anxiety; H4, Moderation effect of social comparison orientation.

Tourists increasingly use social media to narrate their trips, curate impressions, preserve memories, and maintain social ties around leisure experiences (Munar and Jacobsen, 2014; Ghaderi et al., 2024; Chen et al., 2023). Although travel sharing can generate positive outcomes such as social recognition, identity expression, interpersonal connection, and enhanced meaning-making (Wong et al., 2024), it can also become psychologically costly when sharing is organized around audience evaluation and impression management (Liu et al., 2022). Building on research on impression management, social comparison, and tourism satisfaction, we develop a moderated mediation framework that asks when strategic self-presentation in travel sharing is associated with less favorable retrospective trip satisfaction. Specifically, we propose that strategic self-presentation is associated with posting anxiety, that posting anxiety is associated with lower retrospective trip satisfaction, and that social comparison orientation strengthens the association between strategic self-presentation and posting anxiety (Figure 1).

### 2.1 The dual nature of strategic self-presentation in travel sharing

Tourism experiences are socially visible resources that can be used to express identity, signal taste and lifestyle, maintain relationships, and construct meaningful personal memories. Social media provides a convenient stage for such presentation because posts persist over time, can be edited before publication, and are evaluated by an audience that may include both close ties and weak ties (Munar and Jacobsen, 2014). In this sense, strategic self-presentation in travel sharing should not be understood as inherently negative. Travelers may strategically select photos, captions, and narratives in order to communicate the value of an experience, share happiness with others, receive social recognition, or preserve a coherent travel memory. Empirical tourism research similarly suggests that sharing can create social value and enhance satisfaction when it supports connection, recognition, and experience enrichment (Wong et al., 2024).

However, the same self-presentational process may also become psychologically costly when the dominant goal shifts from self-expression to audience-focused impression management. Classic self-presentation theory suggests that anxiety is more likely to arise when individuals strongly desire a favorable impression but are uncertain about whether that impression can be achieved (Schlenker and Leary, 1982; Leary and Kowalski, 1990). Travel content is especially relevant to this process because it often communicates attractiveness, lifestyle quality, social status, and cultural capital. In such contexts, tourists may become concerned not only with whether the trip was meaningful to themselves, but also with whether it appears sufficiently interesting, enviable, or aesthetically impressive to others. Selfies and curated visual narratives illustrate how travel experiences can be transformed into public performances that invite evaluation (Lyu, 2016; Dinhopl and Gretzel, 2016; Elshaer et al., 2024).

Accordingly, the present study does not assume that strategic self-presentation always undermines tourist experience. Rather, it focuses on the condition under which strategic self-presentation may turn from adaptive identity expression into emotionally costly evaluation management. This distinction is important because travel sharing may be beneficial when it supports authentic expression, memory construction, and social connection, but may become detrimental when it is organized around comparison, impression pressure, and anticipated judgment. We, therefore, examine posting anxiety as a psychological mechanism that helps explain when strategic self-presentation is associated with less favorable retrospective trip satisfaction.

### 2.2 Conceptual boundaries among the focal constructs

Because the present model includes several evaluation-related constructs, it is important to clarify their conceptual boundaries. Strategic self-presentation in travel sharing refers to a content-curation and impression-management orientation. It concerns how tourists select, edit, and frame travel content in order to manage how their trip and identity are perceived by others (Michikyan et al., 2015). Posting anxiety, by contrast, refers to an affective response surrounding the act of posting, including apprehension about being judged, misunderstood, ignored, or compared unfavorably (Shabahang et al., 2022). Thus, strategic self-presentation concerns how individuals present travel content, whereas posting anxiety concerns how they feel about making that content socially visible. Social comparison orientation is also conceptually distinct. It refers to a relatively stable tendency to evaluate oneself in relation to others (Gibbons and Buunk, 1999). Although comparison concerns may motivate self-presentation in some contexts, the present study treats social comparison orientation as a dispositional boundary condition. In other words, the model does not assume that social comparison orientation is part of strategic self-presentation or posting anxiety. Rather, it asks whether individuals who are more comparison-oriented experience a stronger association between strategic self-presentation and posting anxiety. The three constructs, therefore, differ in psychological level: strategic self-presentation is a self-presentational orientation, posting anxiety is an affective response, and social comparison orientation is a dispositional vulnerability factor.

### 2.3 Posting anxiety as a separable affective response

Posting anxiety is conceptualized as a distinct affective response to perceived evaluative risk in social media posting. It is not defined as the mere presence of impression-management motives. A tourist may strategically curate travel content without feeling strong anxiety, and another tourist may experience posting anxiety because of uncertainty about feedback or audience interpretation. This distinction is consistent with impression-management theory, which treats self-presentational motivation and self-presentational anxiety as related but separable processes (Schlenker and Leary, 1982; Leary and Kowalski, 1990). In social media contexts, posting anxiety involves apprehension about how content will be received, whether it will be judged, and whether it will invite negative evaluation (Alkis et al., 2017; Shabahang et al., 2022). Travel sharing can make this response especially salient because travel posts often display lifestyle, aesthetics, and social identity. When tourists present trips through carefully selected images or narratives, they may become more sensitive to audience judgment and more concerned about whether the post appears sufficiently interesting or socially valued (Elshaer et al., 2024). Accordingly, the present study treats posting anxiety as a psychological response that may be associated with strategic self-presentation, rather than as a component of strategic self-presentation itself.

Contemporary research on evaluative and appearance-related social media processes further supports this framing. Appearance-related social media consciousness captures users' awareness of how they may be evaluated by online audiences, especially in visually oriented contexts (Di Gesto et al., 2025). Although this work originates in body image research, it highlights a broader process relevant to travel sharing, where photos, selfies, captions, and lifestyle displays may increase audience-focused self-monitoring. Related studies show that focus on self-presentation, feedback-seeking, strategic self-presentation, and upward comparison are relevant to psychological adjustment and wellbeing (Hjetland et al., 2022). Comparison-oriented social media engagement has also been linked to lower self-esteem (Vogel et al., 2014), perceptions that others have happier and better lives (Chou and Edge, 2012), and depressive symptoms through comparison processes (Steers et al., 2014). These findings help situate posting anxiety within a broader literature on evaluative and comparison-oriented social media engagement.

### 2.4 Social comparison processes and their relevance to tourism sharing

Social comparison theory proposes that people evaluate themselves by comparing with others, especially under conditions of uncertainty or self-relevance (Festinger, 1954). Social comparison orientation captures stable individual differences in the extent to which individuals attend to and rely on such comparisons (Gibbons and Buunk, 1999). Social networking sites make comparison cues abundant by presenting curated and positively skewed self-portrayals, and evidence indicates that social comparison on these platforms is a key mechanism linking social media experiences to subjective wellbeing (Verduyn et al., 2020). Meta-analytic evidence further shows that upward comparison on social media is associated with poorer self-evaluations and affective outcomes (McComb et al., 2023). Envy is one pathway through which these processes operate, as comparison with others can elicit discomfort and undermine wellbeing in social networking settings (Krasnova et al., 2015).

In the present framework, social comparison orientation is treated as a boundary condition rather than as the only possible antecedent of strategic self-presentation. We acknowledge that comparison concerns may motivate some individuals to engage in strategic self-presentation in the first place. However, the specific question addressed here is different: given that individuals engage in strategic self-presentation, for whom is this behavior more strongly associated with posting anxiety? Individuals high in social comparison orientation are more likely to interpret travel posts, audience feedback, and others' travel portrayals as information about relative standing. As a result, strategic self-presentation may feel more evaluative and more risky for these individuals, thereby strengthening its association with posting anxiety. This logic positions social comparison orientation as a dispositional vulnerability that shapes when self-presentation becomes psychologically costly.

### 2.5 Retrospective trip satisfaction as a post-trip evaluative outcome

Trip satisfaction reflects tourists' overall evaluation of a travel experience and is shaped by both cognitive assessments and affective responses (Rodríguez del Bosque and San Martín, 2008). In the present study, trip satisfaction is conceptualized specifically as retrospective trip satisfaction, namely a post-trip appraisal of the most recent leisure trip. This clarification is important. We do not assume that tourists wait until they post on social media, or until they receive feedback such as likes and comments, to form an evaluation of their travel experience. Rather, we assume that post-trip satisfaction is an overall retrospective judgment that may be associated with how the trip is remembered, narrated, socially presented, and emotionally processed after or around the travel episode.

Tourism research shows that pleasure and arousal during the experience contribute to satisfaction, indicating that affective states can carry into global judgments (Bigné et al., 2005). Research on documenting experiences also suggests that capturing and sharing moments can shape attention, memory, and later evaluation, depending on whether documentation supports savoring and personal meaning or instead becomes dominated by external evaluation (Diehl et al., 2016; Diehl and Zauberman, 2022). Applied to travel sharing, strategic self-presentation may be associated with lower retrospective trip satisfaction not simply because it interrupts momentary enjoyment during the trip, but because it frames the trip through external standards of how impressive, attractive, or socially desirable the experience appears. When posting anxiety is salient, tourists may ruminate about whether their travel content is good enough, whether others will respond positively, or whether their trip compares favorably with others' experiences. Such evaluative processing may be associated with less favorable post-trip appraisal.

### 2.6 Hypotheses development

#### 2.6.1 H1: Strategic self-presentation and retrospective trip satisfaction

Strategic self-presentation may be negatively associated with retrospective trip satisfaction when travel sharing becomes dominated by audience evaluation rather than personal meaning. When tourists curate travel content primarily to appear interesting, successful, or socially admired, they may evaluate the trip partly through external standards of visibility and comparison. This does not mean that posting determines whether a tourist enjoyed the trip, nor does it imply that satisfaction forms only after online feedback is received. Rather, it suggests that an audience-focused self-presentational orientation may be associated with a less favorable retrospective appraisal because the trip is mentally organized around impression management, adequacy, and social desirability rather than savoring, authenticity, or personal fulfillment. Therefore, greater strategic self-presentation in travel sharing is expected to be associated with lower retrospective trip satisfaction.

**H1**. Strategic self-presentation in travel sharing is negatively associated with retrospective trip satisfaction.

#### 2.6.2 H2: Strategic self-presentation and posting anxiety

Strategic self-presentation may be associated with posting anxiety because audience-oriented impression management increases the perceived stakes of making travel content socially visible. Impression-management theory suggests that anxiety is more likely when individuals strongly desire a favorable impression but remain uncertain about whether that impression will be achieved (Schlenker and Leary, 1982; Leary and Kowalski, 1990). In social media contexts, evaluative concerns and social anxiety tendencies are closely linked to anxiety around posting and audience reactions (Alkis et al., 2017; Shabahang et al., 2022). In travel sharing, where images and narratives are readily compared, strategic self-presentation may, therefore, make posting feel more evaluative, uncertain, and socially risky.

**H2**. Strategic self-presentation in travel sharing is positively associated with posting anxiety.

#### 2.6.3 H3a and H3b: Posting anxiety, retrospective trip satisfaction, and the indirect pathway

Posting anxiety reflects worry about how travel-related content will be judged, misunderstood, ignored, or compared with others' posts. Such anxiety may be associated with lower retrospective trip satisfaction because it introduces negative affect and evaluative rumination into the way the travel experience is processed. Satisfaction integrates affective experience and cognitive evaluation (Rodríguez del Bosque and San Martín, 2008; Bigné et al., 2005); therefore, anxiety surrounding travel sharing may color the post-trip appraisal of the experience. In particular, when tourists focus on whether their posts are sufficiently attractive or socially valued, their retrospective evaluation may become less grounded in personal enjoyment and more tied to perceived audience judgment.

**H3a**. Social media posting anxiety is negatively associated with retrospective trip satisfaction.

Posting anxiety may also serve as a psychological pathway linking strategic self-presentation to retrospective trip satisfaction. When strategic self-presentation makes audience evaluation more salient, tourists may experience greater anxiety about posting. This anxiety, in turn, may be associated with a less favorable retrospective appraisal of the trip. Thus, the proposed indirect pathway does not imply a causal sequence that can be definitively established with cross-sectional data; rather, it specifies a theoretically guided statistical mechanism through which strategic self-presentation is expected to be indirectly associated with retrospective trip satisfaction.

**H3b**. Social media posting anxiety statistically mediates the association between strategic self-presentation in travel sharing and retrospective trip satisfaction.

#### 2.6.4 H4: Moderating role of social comparison orientation

Social comparison orientation captures individual differences in the tendency to compare oneself with others (Gibbons and Buunk, 1999). We acknowledge that social comparison may also function as an antecedent of strategic self-presentation in some contexts, because individuals who frequently compare themselves with others may be motivated to present themselves more favorably. However, the present study focuses on a different theoretical role of social comparison orientation: its role as a boundary condition that shapes when strategic self-presentation becomes emotionally costly. Among individuals who engage in strategic self-presentation, those higher in social comparison orientation are more likely to view travel posts as comparative displays and to interpret audience reactions as signals of relative standing.

Because social media environments increase exposure to curated portrayals and upward comparisons, comparison-oriented users may experience stronger evaluative pressure when they attempt to manage impressions through travel sharing (Verduyn et al., 2020; McComb et al., 2023). For these individuals, strategic self-presentation is more likely to activate concerns about whether their trip appears attractive, enviable, or socially valued. In contrast, individuals low in social comparison orientation may engage in strategic self-presentation without interpreting the posting context as strongly diagnostic of their relative standing. Therefore, social comparison orientation is expected to strengthen the positive association between strategic self-presentation and posting anxiety. We focus on social comparison orientation because it directly matches the comparison-based nature of travel sharing. However, it should be understood as one theoretically central boundary condition, not the only possible moderator. Other evaluative vulnerabilities, such as fear of negative evaluation, contingent self-worth, problematic social media use, self-objectification, and appearance-related social media consciousness, may also shape when strategic self-presentation becomes anxiety-related. These possibilities are discussed as future research directions.

**H4**. Social comparison orientation moderates the association between strategic self-presentation in travel sharing and posting anxiety, such that the association is stronger at higher levels of social comparison orientation.

## 3 Materials and methods

### 3.1 Study design and participants

This study employed a cross-sectional survey design. The target population was adult tourists with a concrete recent experience of travel-related social media sharing, rather than a pre-classified subgroup of “strategic posters.” This distinction is important because strategic self-presentation was treated as a continuous psychological construct, not as an eligibility criterion. Participants could, therefore, vary from relatively authentic, relational, or memory-oriented sharing to more strongly impression-managed sharing. This design allowed us to examine whether variation in strategic self-presentation among actual travel sharers was associated with posting anxiety and retrospective trip satisfaction.

Participants were recruited online. Eligibility criteria were: (a) age ≥ 18 years; (b) completion of at least one leisure trip within the past 3 months; (c) at least one instance of posting travel-related content, such as photos, short videos, or text updates, on social media during or after that trip; and (d) ability to recall the most recent leisure trip and answer the questionnaire with reference to that trip and its related sharing experience. These criteria were used to ensure that all participants had a specific and recent travel-sharing episode on which to base their responses. A total of 490 responses were collected. Twenty-five responses were excluded because they did not meet the eligibility criteria or were incomplete. The final analytic sample consisted of 465 complete responses. Only complete submissions with valid responses on all focal variables and covariates were retained for analysis, and no missing-data imputation was performed.

### 3.2 Procedure

Data were collected using an anonymous online questionnaire. At the beginning of the survey, participants completed eligibility screening items confirming their age, whether they had completed a leisure trip within the past 3 months, and whether they had posted travel-related content on social media during or after that trip. Participants who did not satisfy these criteria were not retained in the analytic sample. Eligible participants were then instructed to think about their most recent leisure trip and the social-media posts they shared about that trip. We emphasized that the study concerned their actual travel-sharing experience and that travel sharing could include authentic recording, memory preservation, relationship maintenance, self-expression, or impression-managed presentation. Participants then completed the study measures in the following order: strategic self-presentation in travel sharing (*X*), social media posting anxiety (*M*), social comparison orientation (*W*), and retrospective trip satisfaction (*Y*). Finally, participants reported background information. When adaptation was needed (e.g., replacing “Facebook” with “social media” and anchoring the wording in the travel-sharing context), standard cross-cultural adaptation procedures were followed, including translation, back-translation, and review for semantic and conceptual equivalence (Beaton et al., 2000).

### 3.3 Measures

All focal constructs were measured using a five-point Likert scale ranging from 1 (*strongly disagree*) to 5 (*strongly agree*). Higher scores indicate higher levels of the construct.

#### 3.3.1 Strategic self-presentation in travel sharing (*X*)

Strategic self-presentation in travel sharing was assessed using the *false-self compare/impress* facet of the Self-Presentation on Facebook Questionnaire (SPFBQ) (Michikyan et al., 2015). This facet captures self-presentational behaviors aimed at comparison and impression management (three items). Items were adapted to the travel-sharing context by referring to participants' posts about their most recent trip and by replacing platform-specific wording with “social media” (e.g., an item reflecting comparing oneself to others when sharing travel content). Item scores were averaged to form a continuous composite, with higher values indicating more strategic self-presentation in travel sharing. This measure was not used to screen participants or to classify them categorically as strategic vs. non-strategic posters. Rather, it measured the degree to which participants' actual travel-sharing behavior involved comparison and impression-management concerns. Lower scores, therefore, indicate less strategic and potentially more authentic, relational, or memory-oriented sharing, whereas higher scores indicate stronger strategic self-presentational tendencies.

#### 3.3.2 Social media posting anxiety (*M*)

Posting anxiety was measured using the Social Media Posting Anxiety Questionnaire (SMPAQ) (Shabahang et al., 2022). The SMPAQ is a brief measure designed specifically to capture anxiety and apprehension about sharing content on social media (six items, one-factor structure). Responses were averaged, with higher scores indicating greater social media posting anxiety.

#### 3.3.3 Social comparison orientation (*W*)

Social comparison orientation was measured with the Iowa–Netherlands Comparison Orientation Measure (INCOM) (Gibbons and Buunk, 1999). The INCOM includes 11 items assessing individuals' dispositional tendency to compare themselves with others across domains. Following standard practice, items were scored and averaged such that higher values indicate stronger social comparison orientation.

#### 3.3.4 Trip satisfaction (*Y*)

Trip satisfaction was assessed using a three-item measure widely used in tourism satisfaction research, operationalizing key components of satisfaction (affective enjoyment, cognitive evaluation, and fulfillment) (Rodríguez del Bosque and San Martín, 2008). Participants rated their agreement with three statements reflecting satisfaction with their most recent trip. Item scores were averaged, with higher scores indicating greater trip satisfaction.

### 3.4 Background information

Participants reported five background variables for descriptive purposes and as covariates in the structural models: (1) gender; (2) age (in years); (3) highest educational attainment; (4) trip type of the recalled leisure trip (domestic overnight, domestic day trip, international, or other); (5) travel-sharing frequency during or after the recalled trip (1–5).

### 3.5 Analytic strategy

Analyses followed a two-step procedure separating the measurement model from the structural model (Anderson and Gerbing, 1988). First, we evaluated the measurement model with confirmatory factor analysis (CFA). Second, we tested the hypothesized moderated mediation model with a latent variable interaction.

#### 3.5.1 Measurement model evaluation

A CFA was conducted to evaluate the distinctiveness of the four latent constructs (*X*, *M*, *W*, and *Y*). Model fit was evaluated using CFI, TLI, RMSEA, and SRMR with commonly used guidelines (Hu and Bentler, 1999). Internal consistency was assessed using Cronbach's α (Dunn et al., 2014).

#### 3.5.2 Data validation, discriminant validity, and common method variance checks

Before testing the structural model, we conducted additional data validation and psychometric checks. To evaluate distributional properties, we inspected skewness and kurtosis for the focal composite scores. No item parceling was used in the CFA or latent-variable models; all item indicators were retained. We inspected item-level indicators for severe univariate non-normality and also examined composite-level skewness and kurtosis for the focal constructs. To supplement Cronbach's alpha, we calculated composite reliability (CR) and average variance extracted (AVE) for each latent construct. Convergent validity was evaluated based on CR and AVE, whereas discriminant validity was examined using the Fornell–Larcker criterion and the heterotrait–monotrait ratio (HTMT) (Fornell and Larcker, 1981; Henseler et al., 2015).

Because all focal variables were measured using self-report Likert scales in a single survey session, we also considered the possibility of common method variance. Procedural steps included anonymous participation, the use of established measures, and instructions that anchored responses to a concrete recent travel-sharing episode. Statistically, we compared the hypothesized four-factor CFA model with a one-factor model in which all items loaded onto a single factor. In addition, we conducted a sensitivity analysis using a common latent factor (CLF) approach to examine whether the hypothesized structural paths remained stable after accounting for shared method variance. These checks were used to evaluate whether common method variance was likely to fully account for the observed associations, although such bias cannot be completely ruled out in a cross-sectional self-report design (Podsakoff et al., 2003; Meier and Reinecke, 2021).

Given the gender imbalance in the sample and the adaptation of established measures to the travel-sharing context, we also conducted a supplementary multi-group CFA to examine measurement invariance across gender. Measurement invariance is especially important for evaluation-related psychological constructs, because apparent group differences may reflect measurement non-equivalence rather than substantive psychological differences (Baroni et al., 2023). We tested configural, metric, and scalar invariance across male and female participants by comparing changes in practical fit indices across increasingly constrained models.

#### 3.5.3 Structural model and latent interaction (LMS)

We estimated the moderated mediation model using the latent moderated structural equations (LMS) approach (Klein and Moosbrugger, 2000; Maslowsky et al., 2015). The structural relations were specified as:


$$\begin{array}{l} M=a_{0}+a_{1}X+a_{2}W+a_{3}\left(X\times W\right)+\varepsilon_{M}, \end{array}$$ (1)

$$\begin{array}{l} Y=b_{0}+b_{1}M+c^{\prime }X+\varepsilon_{Y}. \end{array}$$ (2)

Equations 1, 2 specify the structural relations used in the LMS interaction model. Because LMS models typically do not provide conventional global fit indices (e.g., CFI/TLI/RMSEA/SRMR) due to numerical integration, we evaluated the incremental contribution of the latent interaction by comparing the log-likelihood of a main-effects model (without *X*×*W*) to that of the interaction model (with *X*×*W*) using the log-likelihood difference test (−2Δ*LL*), and by inspecting information criteria (AIC and BIC) (Maslowsky et al., 2015; Marsh et al., 2004).

#### 3.5.4 Inference for indirect and conditional indirect effects

Indirect and conditional indirect effects were computed based on the estimated path coefficients. Uncertainty was quantified using Monte Carlo confidence intervals (MCCI) generated from the sampling distribution implied by the parameter estimates and their covariance matrix (Tofighi and MacKinnon, 2011). Conditional indirect effects were probed at representative values of *W* ($\bar{W}$ and ±1*SD*), and the index of moderated mediation was computed as *a*_3_*b*_1_ (Preacher et al., 2007).

## 4 Results

This section reports the empirical results for the hypothesized moderated mediation model in which strategic self-presentation in travel sharing (*X*) is associated with posting anxiety (*M*), which is associated with retrospective trip satisfaction (*Y*), with social comparison orientation (*W*) moderating the association between *X* and *M*.

### 4.1 Sample characteristics

Table 1 summarizes the demographic profile and travel-related characteristics of the participants. The final sample comprised 465 adult tourists, with women representing approximately two-thirds of the respondents. Most participants were between 18 and 34 years of age, and the modal education level was a bachelor's degree. Regarding the recalled trip, domestic overnight travel was most common, followed by international travel, whereas domestic day trips and other trip types were less frequent. Participants reported moderate to relatively frequent travel-sharing activity on social media during or after the recalled trip.

**Table 1:** Sample characteristics.

Variable	Category	*n* (%) or *M*±*SD*
Gender	Male	163 (35.1%)
	Female	302 (64.9%)
Age	18–24	144 (31.0%)
	25–34	191 (41.1%)
	35–44	84 (18.1%)
	45+	46 (9.9%)
Education	High school or below	22 (4.7%)
	Associate	51 (11.0%)
	Bachelor	273 (58.7%)
	Master	94 (20.2%)
	Doctorate or above	25 (5.4%)
Trip type	Domestic overnight	235 (50.5%)
	Domestic day trip	82 (17.6%)
	International	131 (28.2%)
	Other	17 (3.7%)
Travel-sharing frequency	Scale (1–5)	3.72 ± 0.91

Travel-sharing frequency is coded from 1 to 5, with higher scores indicating more frequent posting (or planning to post) travel-related content during the recalled trip.

### 4.2 Preliminary analyses

Table 2 presents the descriptive statistics, internal consistency estimates, and bivariate associations among the focal constructs. Mean levels were moderate to moderately high across variables, with retrospective trip satisfaction showing the highest average score. Internal consistency was satisfactory for all measures, with Cronbach's alpha values ranging from 0.821 to 0.892.

**Table 2:** Descriptive statistics, reliability, and correlations among focal constructs.

Variable	*Mean*	*SD*	α	(1)	(2)	(3)	(4)
(1) Strategic self-presentation (*X*)	3.422	0.884	0.864	1.000			
(2) Social media posting anxiety (*M*)	3.148	0.921	0.892	0.284***	1.000		
(3) Social comparison orientation (*W*)	3.581	0.735	0.821	0.115*	0.192***	1.000	
(4) Retrospective trip satisfaction (*Y*)	3.824	0.792	0.853	–0.106*	–0.213***	–0.042	1.000

^*^p < 0.05, ^***^p < 0.001. *Mean* and SD represent the mean and standard deviation, respectively. α denotes Cronbach's alpha.

Consistent with the proposed framework, strategic self-presentation in travel sharing was positively associated with posting anxiety, indicating that individuals who reported stronger strategic self-presentational tendencies also tended to experience greater anxiety about posting. Posting anxiety was negatively related to retrospective trip satisfaction, suggesting that higher anxiety surrounding travel-related posting corresponded to lower satisfaction with the recalled trip. Social comparison orientation showed small positive correlations with both strategic self-presentation and posting anxiety, whereas its association with retrospective trip satisfaction was not statistically significant. In addition, strategic self-presentation showed a small negative correlation with retrospective trip satisfaction.

### 4.3 Confirmatory factor analysis

We conducted a confirmatory factor analysis to examine whether the four focal constructs were empirically distinguishable. As shown in Table 3, the hypothesized four-factor model provided an acceptable representation of the data, with fit indices indicating good overall fit. In contrast, the three-factor model that combined strategic self-presentation and posting anxiety fit the data substantially worse, suggesting that these constructs should not be treated as a single factor. The one-factor model yielded poor fit, further supporting the distinctiveness of the measured constructs and reducing concern that the observed associations were driven by a single common factor.

**Table 3:** Confirmatory factor analysis (CFA) model fit indices.

Model specification	χ^2^	*df*	CFI	TLI	RMSEA	SRMR
Four-factor model: *X, M, W, Y*	428.152	224	0.942	0.934	0.054	0.048
Three-factor model: (*X*+*M*), *W, Y*	982.463	227	0.784	0.759	0.112	0.096
One-factor model: all items → one factor	1,854.921	230	0.536	0.491	0.184	0.142

*X* = Strategic self-presentation; *M* = Social media posting anxiety; *W* = Social comparison orientation; *Y* = Trip satisfaction.

### 4.4 Data validation and common method variance checks

To address data distribution and psychometric validation, we conducted additional checks of normality, reliability, convergent validity, and discriminant validity. No item parceling was used in the CFA or LMS analyses; all item indicators were retained in the latent-variable models. Inspection of the item-level indicators showed no severe univariate non-normality. As shown in Table 3, the composite-level skewness values ranged from −0.542 to 0.187, and kurtosis values ranged from −0.341 to 0.245. These values indicated no severe departure from univariate normality. In addition, the structural models were estimated using robust maximum likelihood, which further reduced sensitivity to moderate non-normality.

The measurement validation results also supported reliability and construct validity. Composite reliability values ranged from 0.828 to 0.896, exceeding the commonly used threshold of 0.70. Average variance extracted values ranged from 0.521 to 0.689, exceeding the recommended threshold of 0.50 and supporting convergent validity. Discriminant validity was supported in two ways. First, the square root of AVE for each construct ranged from 0.722 to 0.830, exceeding the corresponding inter-construct correlations reported in Table 2. Second, the maximum HTMT value was 0.324, well-below conservative and liberal thresholds of 0.85 and 0.90, respectively. These results, together with the CFA model comparisons reported above, support the empirical distinctiveness of the four focal constructs.

Common method variance was also considered because all focal variables were collected through self-report in a single survey. The poor fit of the one-factor CFA model reported in Table 3 (*CFI* = 0.536, *TLI* = 0.491, *RMSEA* = 0.184, and *SRMR* = 0.142) suggests that a single common factor did not adequately represent the data. However, because one-factor CFA comparisons alone cannot rule out common method bias, we conducted an additional CLF sensitivity analysis. As shown in Table 4, the hypothesized structural coefficients were slightly attenuated after including the common latent method factor, but all focal paths retained the same direction and remained statistically significant. These results suggest that shared method variance is unlikely to fully account for the hypothesized associations. Nevertheless, because the data are cross-sectional and self-reported, common method variance cannot be completely ruled out, and the results should be interpreted as theoretically guided statistical associations rather than causal effects (Table 5).

**Table 4:** Sensitivity analysis of structural path estimates with a common latent method factor.

	Original LMS model		CLF-adjusted LMS model	
Path	Unstd. *B*	*p*-value	Unstd. *B*	*p*-value
*X*→*M* (Strategic self-presentation → Posting anxiety)	0.245	0.021	0.223	0.031
*W*→*M* (Social comparison orientation → Posting anxiety)	0.198	0.016	0.181	0.024
*X*×*W*→*M* (Latent interaction effect)	0.186	0.010	0.171	0.014
*M*→*Y* (Posting anxiety → Retrospective trip satisfaction)	–0.218	< .001	–0.196	0.002
*X*→*Y* (Direct path)	–0.106	0.046	–0.098	0.048

**Table 5:** Normality, reliability, convergent validity, and discriminant validity statistics.

Construct	Skewness	Kurtosis	CR	AVE	$\sqrt{\text{AVE}}$	Max HTMT
Strategic self-presentation (*X*)	–0.214	–0.341	0.869	0.689	0.830	0.324
Posting anxiety (*M*)	0.187	–0.108	0.896	0.593	0.770	0.324
Social comparison orientation (*W*)	–0.103	0.245	0.828	0.521	0.722	0.228
Retrospective trip satisfaction (*Y*)	–0.542	0.119	0.858	0.668	0.817	0.246

*N* = 465. CR, composite reliability; AVE, average variance extracted; HTMT, heterotrait–monotrait ratio. Skewness and kurtosis are reported for composite scores. Max HTMT represents the highest HTMT ratio between the focal construct and any other construct in the model.

We also examined whether the adapted measures functioned equivalently across gender groups. As shown in Table 6, the configural invariance model showed acceptable fit, indicating that the same four-factor structure was supported among male and female participants. Imposing equality constraints on factor loadings produced only trivial changes in model fit (Δ*CFI* = −0.001, Δ*RMSEA* = −0.001, Δ*SRMR* = 0.003), supporting metric invariance. Further constraining item intercepts also produced small changes in fit (Δ*CFI* = −0.003, Δ*RMSEA* = 0.002, Δ*SRMR* = 0.003), supporting scalar invariance. These results suggest that the adapted measures showed strong measurement invariance across gender groups in the present sample.

**Table 6:** Measurement invariance across gender.

Model	χ^2^	*df*	CFI	TLI	RMSEA	SRMR	*ΔCFI*	*ΔRMSEA*	*ΔSRMR*
Model 1: Configural invariance	461.375	448	0.944	0.936	0.053	0.046	–	–	–
Model 2: Metric invariance	482.114	467	0.943	0.937	0.052	0.049	–0.001	–0.001	+0.003
Model 3: Scalar invariance	514.652	486	0.940	0.936	0.054	0.052	–0.003	+0.002	+0.003

*N*_male_ = 163, *N*_female_ = 302. Δ comparison is made relative to the preceding less constrained model. Changes in practical fit indices support the retention of strong (scalar) measurement invariance across gender groups.

### 4.5 Structural model and hypothesis testing

We examined the hypothesized moderated mediation model. Posting anxiety (*M*) was regressed on *X*, *W*, and their latent interaction, and trip satisfaction (*Y*) was regressed on *M* and *X*. Background variables were included as covariates in both equations.

#### 4.5.1 Model estimation and LMS specific model comparison

To determine whether including the latent interaction improved the model, we compared a main effects model without the interaction term (Model 0) with the LMS interaction model (Model 1). As shown in Table 7, Model 1 yielded a lower −2 log likelihood than Model 0 (−2*LL* = 8, 444.612 vs. 8, 452.184). Information criteria also favored the interaction model, with Model 1 showing smaller values for both AIC (8, 598.612 vs. 8, 604.184) and BIC (8, 917.565 vs. 8, 918.992). Consistent with these patterns, the MLR corrected log likelihood difference test indicated that adding the latent interaction provided a statistically meaningful improvement in fit, −2Δ*LL* = 6.824 with Δ*df* = 1, *p* = 0.009. Taken together, these results support retaining the latent interaction term in subsequent hypothesis tests.

**Table 7:** Model comparison for the structural models.

Model	−2*LL*	*k*	SCF	AIC	BIC	Notes
Model 0: Baseline with covariates	8,452.184	76	1.082	8,604.184	8,918.992	Main effects only
Model 1: LMS with covariates	8,444.612	77	1.104	8,598.612	8,917.565	With *X*×*W*

−2*LL*, −2 log-likelihood; *k*, number of free parameters; SCF, scaling correction factor under MLR. Background variables were included as covariates. Satorra-Bentler scaled difference test: −2Δ*LL* = 6.824, Δ*k* = 1, *p* = 0.009.

#### 4.5.2 Parameter estimates in the LMS interaction model

Table 8 reports the parameter estimates from the LMS interaction model. Strategic self-presentation in travel sharing was positively related to posting anxiety, *B* = 0.245, *p* = 0.021, and social comparison orientation was also positively related to posting anxiety, *B* = 0.198, *p* = 0.016. Most importantly, the latent interaction term was significant, *B* = 0.186, *p* = 0.010, indicating that the association between strategic self-presentation and posting anxiety was stronger at relatively higher levels of social comparison orientation. Posting anxiety was negatively associated with retrospective trip satisfaction, *B* = −0.218, *p* < 0.001. In addition, strategic self-presentation showed a small but significant direct association with lower retrospective trip satisfaction, *B* = −0.106, *p* = 0.046, suggesting that the proposed indirect pathway captured a meaningful part of the relation while leaving room for additional mechanisms beyond posting anxiety. Regarding the background variables, travel sharing frequency was associated with higher posting anxiety, whereas the remaining covariates were not statistically related to posting anxiety. None of the covariates emerged as significant predictors of trip satisfaction in the full model.

**Table 8:** Structural path estimates for the LMS interaction model.

Path	Unstd. *B*	S.E.	*z*	*p*
Hypothesized paths				
*X*→*M* (*a*_1_)	0.245	0.106	2.311	0.021
*W*→*M* (*a*_2_)	0.198	0.082	2.415	0.016
*X*×*W*→*M* (*a*_3_)	0.186	0.072	2.583	0.010
*M*→*Y* (*b*_1_)	–0.218	0.058	–3.759	< 0.001
*X*→*Y* (*c*′)	–0.106	0.053	–2.000	0.046
Covariates predicting posting anxiety (*M*)				
Gender (Female = 1)	0.062	0.041	1.512	0.131
Age	–0.014	0.011	–1.273	0.203
Education	0.035	0.028	1.250	0.211
Domestic day trip^a^	0.042	0.036	1.167	0.243
International trip^a^	0.076	0.048	1.583	0.113
Other trip type^a^	0.015	0.039	0.385	0.700
Travel-sharing frequency	0.104	0.045	2.311	0.021
Covariates predicting retrospective trip satisfaction (*Y*)				
Gender (Female = 1)	0.021	0.032	0.656	0.512
Age	0.005	0.008	0.625	0.532
Education	–0.012	0.025	–0.480	0.631
Domestic day trip^a^	0.018	0.029	0.621	0.535
International trip^a^	–0.054	0.038	–1.421	0.155
Other trip type^a^	0.008	0.027	0.296	0.767
Travel-sharing frequency	–0.034	0.031	–1.097	0.273

*N* = 465. *B*, Unstandardized coefficient; *SE*, Robust standard error.

^a^Reference category is Domestic overnight trip.

Covariates (age, education, and travel-sharing frequency) were mean-centered; covariates (gender, trip type) were dummy-coded and not centered.

#### 4.5.3 Simple slopes for the moderated path

To clarify the nature of the interaction, we examined the conditional association between strategic self-presentation and posting anxiety at representative values of the continuous moderator. Specifically, social comparison orientation was probed at one standard deviation below and above the mean of *W* using the expression:


$$\frac{\partial M}{\partial X}=a_{1}+a_{3}W.$$

**Figure 2:**
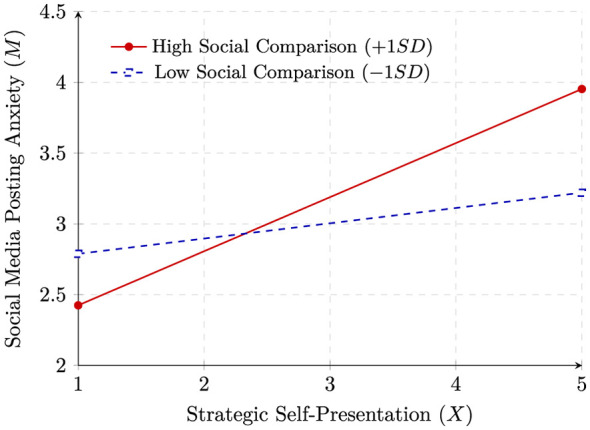
The moderating role of social comparison orientation (*W*) on the relationship between strategic self-presentation (*X*) and posting anxiety (*M*).

As shown in Table 9, the association between *X* and *M* was small and not statistically significant at one standard deviation below the mean of social comparison orientation, *B* = 0.108, *p* = 0.261. In contrast, at one standard deviation above the mean, strategic self-presentation was positively associated with posting anxiety, *B* = 0.382, *p* < 0.001. Figure 2 illustrates this pattern, showing a relatively flatter slope at lower representative values of social comparison orientation and a steeper increase in posting anxiety as strategic self-presentation rises at higher representative values of social comparison orientation.

**Table 9:** Simple slopes of *X* predicting *M* at levels of *W*.

Moderator level	Slope (*a*_1_+*a*_3_*W*)	S.E.	*z*	*p*
Low *W* (−1*SD*)	0.108	0.096	1.125	0.261
High *W* (+1*SD*)	0.382	0.094	4.064	< .001

Low *W* and high *W* refer to representative values of the continuous moderator, defined as one standard deviation below the mean (−0.735) and one standard deviation above the mean (+0.735), respectively. These values are used only to probe the continuous interaction and do not represent categorical groups.

### 4.6 Indirect effects, conditional indirect effects, and index of moderated mediation

We next examined whether strategic self-presentation was indirectly associated with retrospective trip satisfaction through posting anxiety, and whether this indirect association varied across representative values of social comparison orientation. Conditional indirect effects were computed as (*a*_1_+*a*_3_*W*)*b*_1_, and uncertainty was summarized using Monte Carlo confidence intervals.

As reported in Table 10, the indirect association depended on social comparison orientation. At one standard deviation below the mean of social comparison orientation, the estimated indirect effect was small and not statistically different from zero, *B* = −0.024, with a confidence interval that included zero. At the mean level of social comparison orientation, the indirect effect was negative and statistically significant, *B* = −0.053, 95% MC CI [−0.112, −0.011]. At one standard deviation above the mean, the indirect effect was strongest, *B* = −0.083, 95% MC CI [−0.152, −0.032]. This pattern indicates that the indirect association between strategic self-presentation and retrospective trip satisfaction through posting anxiety becomes more pronounced at relatively higher values of social comparison orientation.

**Table 10:** Conditional indirect effects and index of moderated mediation.

Effect	Estimate	MC SE	95% MC CI
Conditional indirect effect: **X*→*M*→*Y**			
Indirect at *W* = −−1*SD*	–0.024	0.022	[–0.071, 0.015]
Indirect at $W=\bar{W}$	-0.053	0.026	[–0.112, –0.011]
Indirect at *W* = +1*SD*	–0.083	0.031	[–0.152, –0.032]
Index of moderated mediation			
Index (*a*_3_×*b*_1_)	–0.041	0.018	[–0.082, –0.012]

Unstandardized indirect effects calculated as (*a*_1_+*a*_3_*W*) × *b*_1_. 95% MC CI, 95% Monte Carlo Confidence Interval (based on 20,000 simulations). Bold estimates indicate statistical significance (CI does not include zero). *W*, Social comparison orientation and was mean-centered; therefore, $\bar{W}=0$.

**Figure 3:**
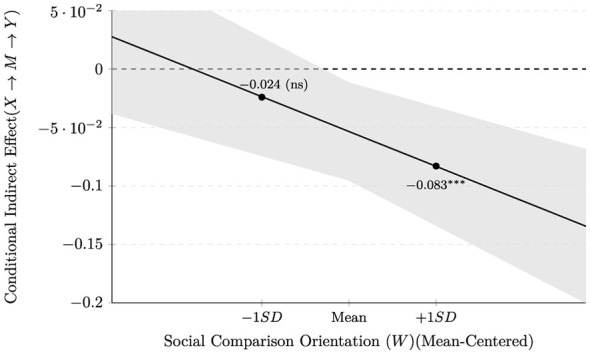
Conditional indirect effect of strategic self-presentation (*X*) on retrospective trip satisfaction (*Y*) through posting anxiety (*M*) as a function of social comparison orientation (*W*).

The index of moderated mediation was also statistically significant, *B* = −0.041, 95% MC CI [−0.082, −0.012], providing direct evidence that the indirect effect varies as a function of social comparison orientation. Figure 3 illustrates the same trend, showing increasingly negative conditional indirect effects at relatively higher values of social comparison orientation.

## 5 Discussion

The present study examined whether strategic self-presentation in travel sharing is associated with retrospective trip satisfaction through social media posting anxiety, and whether this association differs by social comparison orientation. The results indicate a theoretically coherent pattern of associations. Strategic self-presentation was positively related to posting anxiety, posting anxiety was negatively related to retrospective trip satisfaction, and the indirect association between strategic self-presentation and retrospective trip satisfaction through posting anxiety was stronger at higher levels of social comparison orientation. Because the data are cross-sectional, these findings should not be interpreted as evidence that strategic self-presentation causes posting anxiety or reduces satisfaction. Rather, they identify statistically supported associations among theoretically distinct constructs.

### 5.1 Strategic self-presentation and posting anxiety in the travel-sharing context

A central implication of our findings is that posting anxiety can be understood as an affective cost of strategic self-presentation. Classic accounts of self-presentation emphasize that when individuals aim to influence others' impressions, they become more vigilant to social evaluation and to cues that signal acceptance or rejection (Schlenker and Leary, 1982; Leary and Kowalski, 1990). In social media environments, this vigilance is often amplified because the audience is potentially broad, heterogeneous, and partly invisible, which can raise uncertainty about how content will be interpreted (Marwick and boyd, 2011). This structural feature of mediated settings aligns with the motivational view that people approach social media partly to meet interpersonal needs, while simultaneously managing the risks that come with visibility and judgment (Nadkarni and Hofmann, 2012).

Within this framework, the positive association between strategic self-presentation and posting anxiety suggests that impression management during travel sharing may invite a heightened fear of negative evaluation. Domain-specific evidence indicates that posting anxiety is closely tied to concerns about being judged, misunderstood, or socially sanctioned for what one shares (Shabahang et al., 2022). Related work on online self-disclosure similarly shows that evaluative concerns can inhibit or distort disclosure processes, and that fear of evaluation is a psychologically meaningful driver of how people manage what they reveal online (Zeng and Zhu, 2021). In the travel-sharing context, strategic self-presentation likely directs attention toward standards of attractiveness, excitement, or social desirability embedded in the local posting culture. When the posting goal shifts from documenting an experience to curating an image, the psychological meaning of posting may change from communication to performance, which plausibly elevates anxiety.

The association we observed also fits with evidence that social media can shape wellbeing by altering how people interpret their own lives relative to others. Experimental and longitudinal work has linked social media engagement to changes in affect and wellbeing, in part by fostering evaluative and comparison-based processing (Kross et al., 2013; Verduyn et al., 2015). While travel sharing is a distinctive context, it may be especially prone to such evaluative processing because travel content is often normatively positive and selectively presented. This kind of positivity can raise expectations about what a good trip should look like and may create pressure to narrate the experience in ways that meet perceived standards, thereby contributing to posting anxiety.

### 5.2 Posting anxiety as a mechanism linked to retrospective trip satisfaction

The negative association between posting anxiety and retrospective trip satisfaction suggests that posting-related anxiety is not merely an online experience, but is also linked to how participants appraise a recent offline travel episode. Satisfaction judgments are shaped by affective states that occur during an experience and by how those states are integrated into memory-based evaluations. When posting anxiety is salient, it may reduce satisfaction by increasing self-focused attention, narrowing cognitive bandwidth, and encouraging ruminative evaluation of one's social standing. Such processes are consistent with broader psychological accounts in which evaluative anxiety disrupts immersion and attenuates positive affect (Schlenker and Leary, 1982). In mediated contexts, anxiety may also persist after the event, as individuals anticipate feedback, monitor reactions, and reinterpret the meaning of their shared content (Shabahang et al., 2022).

A complementary perspective is that certain media-related behaviors can shift how people allocate attention during meaningful moments. Research on documenting experiences suggests that orienting toward capture and presentation can change experience quality and later evaluation. For example, taking photos can sometimes increase enjoyment by focusing attention on positive aspects, yet it can also introduce tradeoffs when capture becomes a goal in itself (Diehl et al., 2016). More recent integrative work argues that the effects of capturing life are heterogeneous and depend on motives and context, including whether capture supports savoring or instead crowds it out (Diehl and Zauberman, 2022). In our model, posting anxiety reflects a motivational climate in which capture and sharing are intertwined with evaluation, which may make the costs of capture more likely to surface. Under such conditions, anxiety may reduce the extent to which tourists can remain psychologically present, and it may bias retrospective evaluation toward what was posted, how it might be judged, and whether the shared narrative matches perceived norms.

This interpretation aligns with research showing that passive or evaluative social media engagement can undermine momentary affect and broader wellbeing (Verduyn et al., 2015). It also resonates with evidence that social media feedback is psychologically meaningful. Social endorsement cues such as likes can engage social reward processes and may thereby increase the motivational stakes of posting (Sherman et al., 2016). In the context of travel sharing, where posts often serve as public markers of lifestyle and identity, feedback cues may become especially diagnostic for self-worth or social status, amplifying anxiety and shaping how the trip is evaluated.

### 5.3 Why social comparison orientation strengthens the self-presentation to anxiety link

The moderation results underscore that the psychological cost of strategic self-presentation is not uniform. Social comparison theory proposes that individuals use others as standards for self-evaluation, particularly under uncertainty (Festinger, 1954). Social comparison orientation captures stable individual differences in the tendency to engage in such comparisons (Gibbons and Buunk, 1999). People high in social comparison orientation may be more likely to interpret travel sharing as a comparison opportunity and to treat audience reactions as diagnostic feedback. In turn, the same self-presentational behavior may feel more consequential and more risky, which plausibly increases anxiety.

This account is consistent with social media research showing that comparison processes are central to how people experience mediated environments. Reviews highlight that social media afford frequent exposure to others' curated successes, thereby facilitating upward comparisons and comparison-related emotions (Appel et al., 2016; Verduyn et al., 2020). Empirical work indicates that social media use is associated with social comparison and self-evaluative outcomes, including lower self-esteem among some users (Vogel et al., 2014). Moreover, work on comparison and envy suggests that exposure to upward targets can evoke negative self-evaluations and affective discomfort, particularly when users perceive others as better off (Chou and Edge, 2012; Krasnova et al., 2015). Recent synthesis further emphasizes that social comparison and envy are not inevitable outcomes of social media exposure, but become more likely under certain motives and person-specific susceptibilities (Meier and Johnson, 2022).

Our conditional indirect effects are consistent with this person-specific view. When social comparison orientation was low, strategic self-presentation was not strongly related to posting anxiety, and the indirect association with trip satisfaction was correspondingly weak. When social comparison orientation was high, the path from strategic self-presentation to posting anxiety was stronger and the conditional indirect effect was more negative. These patterns align with broader arguments that the psychological consequences of social media depend on what people do on platforms and why they do it (Valkenburg, 2022). Meta-analytic work focused on positive wellbeing outcomes similarly finds that broad measures of social media use tend to yield near-zero associations, whereas specific activities and experiences show more differentiated relations with wellbeing (Marciano et al., 2024). In this sense, our findings contribute evidence that a specific sharing motive, namely strategic self-presentation, combines with a specific disposition, namely social comparison orientation, to predict a specific affective state, namely posting anxiety, which then shapes satisfaction with a concrete life episode.

### 5.4 Theoretical contributions

The theoretical contribution of this study is not that self-presentation and anxiety are newly connected. This relation is already well-established in self-presentation and impression-management theories, which emphasize that evaluative uncertainty and concern about others' impressions can generate anxiety (Schlenker and Leary, 1982; Leary and Kowalski, 1990). Rather, our contribution is to specify how this established principle operates in social media travel sharing, when it becomes more pronounced, and how it is associated with retrospective appraisal of an offline leisure experience.

First, the study refines impression-management theory by locating strategic self-presentation in a concrete travel-sharing context. Travel sharing is not merely generic online self-presentation; it transforms an offline leisure episode into a public narrative of lifestyle, taste, social connection, and identity. Strategic self-presentation may be adaptive when it supports identity expression, memory construction, and social connection, but it may become costly when it shifts toward audience-focused evaluation management. This distinction helps clarify when self-presentation moves from adaptive expression to anxiety-related impression management (Leary and Kowalski, 1990).

Second, the study specifies posting anxiety as a proximal and domain-specific affective mechanism. Rather than treating anxiety as a general outcome of social media use, we focus on anxiety surrounding the act of posting travel-related content, including concerns about being judged, ignored, misunderstood, or unfavorably compared. This responds to calls to move beyond broad usage indicators and examine specific activities, motives, and psychological pathways in social media and wellbeing research (Verduyn et al., 2015; Valkenburg, 2022).

Third, the study clarifies social comparison orientation as a boundary condition. Social comparison theory suggests that individuals differ in how strongly they rely on comparison information when evaluating themselves (Gibbons and Buunk, 1999). Our model does not deny that comparison concerns may also motivate strategic self-presentation. Instead, it shows that among individuals who engage in strategic self-presentation, those higher in social comparison orientation may be more likely to experience posting as evaluative and anxiety-provoking.

Finally, the study links online impression-management processes to retrospective trip satisfaction. Whereas social media research often examines general wellbeing and tourism research often emphasizes the positive value of sharing, our findings identify a more specific pattern connecting strategic self-presentation, posting anxiety, social comparison orientation, and post-trip appraisal. This contribution is consistent with recent reviews emphasizing that social media effects are heterogeneous and depend on specific activities, mechanisms, and user characteristics (Meier and Reinecke, 2021; Godard and Holtzman, 2024). Because the data are cross-sectional, this contribution is framed in associational rather than causal terms.

### 5.5 Practical implications for meaningful travel sharing

The findings suggest that practical efforts should not discourage social media travel sharers from sharing travel experiences, but should help shift the function of sharing from impression management to meaningful self-expression. At the individual level, these users may benefit from brief pre-posting reflection, such as asking whether a post is intended to preserve a personally meaningful memory or mainly to prove that the trip appears attractive to others. Such intention checks may redirect attention from audience judgment toward personal meaning, authenticity, and enjoyment, consistent with the view that capturing and sharing experiences are more beneficial when they support savoring and connection rather than performance (Diehl and Zauberman, 2022).

At the intervention level, digital wellbeing or travel-related media literacy programs could encourage meaning-focused posting strategies. Travelers can be guided to write captions about what they learned, what felt memorable, or why a moment mattered, rather than focusing only on whether the post looks impressive. Delayed posting, private travel journals, or selective-audience sharing may also reduce immediate comparison pressure and posting anxiety.

At the platform level, design features that reduce evaluative salience may be useful. Social endorsement cues can increase the motivational stakes of posting (Sherman et al., 2016), while context collapse may increase uncertainty about audience interpretation (Marwick and boyd, 2011). Therefore, options such as hiding like counts, strengthening close-friends sharing, or providing reflective prompts before posting could help users treat travel sharing less as a public performance and more as self-expression and memory construction. These suggestions are consistent with calls for activity-specific and mechanism-focused approaches to digital wellbeing (Valkenburg, 2022; Godard and Holtzman, 2024).

### 5.6 Limitations and future research directions

Several limitations should be acknowledged. First, the study used a cross-sectional survey design. Therefore, the moderated mediation model should be interpreted as a theoretically guided pattern of associations rather than evidence of causal or temporal processes. Future research could use longitudinal, experimental, or experience-sampling designs to examine how travel sharing, posting anxiety, social comparison, and trip appraisal unfold before, during, and after a trip.

Second, all focal variables were measured using self-report scales in a single survey session. Although the CFA results, measurement validation checks, and CLF sensitivity analysis suggest that common method variance is unlikely to fully explain the findings, it cannot be completely ruled out. Consistent with methodological recommendations in social media and wellbeing research (Meier and Reinecke, 2021), future studies could combine survey measures with longitudinal designs, experience-sampling methods, and behavioral trace data, such as actual posting frequency, audience scope, posting delay, likes, comments, or privacy settings.

Third, the sample consisted of adult tourists who had recently shared travel-related content on social media, with a relatively high proportion of young, female, and highly educated participants. The findings should, therefore, be generalized primarily to social media travel sharers rather than to all tourists or all social media users. Moreover, because eligibility required at least one travel-related post, the sample may have overrepresented individuals who were relatively comfortable with social media self-presentation and underrepresented tourists who avoid posting, post rarely, or only consume travel content passively. This selection feature may have restricted variability in strategic self-presentation and posting anxiety. Although we conducted supplementary measurement invariance testing across gender, future research with larger and more balanced subgroup samples should test the model among more diverse age groups, education levels, travel types, platform environments, and cultural contexts.

Finally, this study focused on an anxiety-related cost pathway and measured retrospective trip satisfaction. Future research should examine other evaluative vulnerability factors that may moderate the psychological cost of strategic travel sharing, including fear of negative evaluation, contingent self-worth, problematic social media use, self-objectification, and appearance-related social media consciousness. Future work could also examine positive and negative pathways together, including self-expression, social connection, memory construction, social recognition, savoring, and evaluative anxiety. Such research would further clarify when strategic self-presentation supports meaningful sharing and when it becomes dominated by comparison and evaluative pressure.

## 6 Conclusion

The present findings suggest that, among adult social media travel sharers, strategic self-presentation in travel-related sharing is associated with posting-related psychological costs. Participants who approached sharing as a means of managing impressions tended to report greater posting anxiety, and this anxiety was linked to lower retrospective satisfaction with their most recent trip. This pattern was most pronounced among individuals with relatively higher social comparison orientation, suggesting that dispositional sensitivity to interpersonal comparison may amplify the emotional burden of self-presentational sharing. Taken together, the study connects theories of self-presentation and social comparison to an episode-level evaluative outcome, while emphasizing that the findings represent cross-sectional associations within a sample of recent travel sharers rather than causal effects generalizable to all tourists.

## Data Availability

The original contributions presented in the study are included in the article/supplementary material, further inquiries can be directed to the corresponding author/s.
